# Analysis of Novel DNA Adducts Derived from Acetaldehyde

**DOI:** 10.3390/biom15060878

**Published:** 2025-06-16

**Authors:** Yuuki Betsuyaku, Mina Motohashi, Akira Sassa, Takeji Takamura-Enya, Yukari Totsuka

**Affiliations:** 1 Department of Chemistry, Kanagawa Institute of Technology, Atsugi-shi, Kanagawa 243-0292, Japan; bababe.yb20@gmail.com (Y.B.); takamura@chem.kanagawa-it.ac.jp (T.T.-E.); 2 Division of Carcinogenesis & Prevention, National Cancer Center Research Institute, Chuo-ku, Tokyo 104-0045, Japan; 3 Laboratory of Environmental Toxicology and Carcinogenesis, School of Pharmacy, Nihon University, Funabashi-shi, Chiba 274-8555, Japan; namosu18@yahoo.co.jp; 4 Graduate School of Science, Chiba University, Inage-ku, Chiba 263-8522, Japan; a-sassa@chiba-u.jp; 5 Department of Environmental Health Sciences, Hoshi University, Shinagawa-ku, Tokyo 142-8501, Japan

**Keywords:** DNA adductome, mutation signature, SBS16, acetaldehyde, *N*^6^-ethyl-dA, *N*^1^-oxydiethylidene-dA

## Abstract

Alcohol consumption is a known risk factor for esophageal and liver cancers. Recently, it was reported that mutation signatures characterized by T:A to C:G mutations (SBS16), which are suggested to be associated with alcohol intake, are frequently detected in esophageal, liver, and stomach cancers among the Japanese population. However, the scientific evidence linking alcohol consumption to SBS16 remains lacking. Acetaldehyde (AA), a carcinogenic metabolite of alcohol, is considered a key contributor to alcohol-related cancer development. Although the guanine adducts associated with alcohol exposure have been reported as part of its carcinogenic mechanism, an adenine adduct, *N*^6^-ethyl-deoxyadenosine (*N*^6^-ethyl-dA), a potential contributor to the SBS16 mutation pattern, was recently identified using a mass spectrometry-based DNA adductome approach. However, the mutagenicity assessment of *N*^6^-ethyl-dA using primer extension assays and the *supF* gene mutation test showed that this adenine adduct is not mutagenic. To identify another candidate as a driver adduct for SBS16, a DNA adductome approach was conducted, leading to the identification of a novel adenine adduct, 3-(2′-deoxyribos-1′-yl)-7,9-dimethyl-3,9-dihydro-7*H*-[1,3,5]oxadiazino[4,3-*i*]purine (*N*^1^-oxydiethylidene-dA), in which two AA molecules are bound to an adenine base. Moreover, *N*^1^-oxydiethylidene-dA was detected in mouse livers, and its levels increased following ethanol administration, suggesting that alcohol may contribute to SBS16 induction via the formation of *N*^1^-oxydiethylidene-dA.

## 1. Introduction

Drinking alcohol is a known cause of cancer in humans. According to the Global Burden of Cancer in 2020 Attributable to Alcohol Consumption study, over 740,000 cancer cases were linked to alcohol consumption [1]. The International Agency for Research on Cancer has classified alcohol as a Group 1 human carcinogen and identified it as a cause of breast, colorectal, laryngeal, liver, esophageal, oral, and pharyngeal cancers [2,3]. A systematic literature review on alcohol consumption and cancer risk analyzed research articles published between 2002 and 2012 [4]. This review noted that most individuals who smoke also consume alcohol, making adjustment for tobacco use essential to avoid misattributing the cancer risk to alcohol alone. Furthermore, the review highlighted that the amount of alcohol consumed is more significant than the type of beverage, concluding that after adjusting for tobacco use, from moderate to heavy alcohol consumption increases the risk of cancers of the esophagus, the stomach, the larynx, the pancreas, and the breast. In contrast to Western countries, one study reported a difference in risk magnitude between men and women in the Japanese population [5]. While a positive association was found between alcohol consumption and the total cancer risk, no clear association was observed for women. The authors speculated that this was due to the low number of regular or heavy female drinkers, rather than a true sex-based difference in disease susceptibility. A recent study in the Korean population reported similar findings [6].

The carcinogenic mechanism of alcohol is believed to be linked to the in vivo formation of its metabolite, the carcinogen acetaldehyde (AA) [2,3]. The majority of alcohol metabolism is facilitated by alcohol dehydrogenases and aldehyde dehydrogenases (ALDHs), while the genes of both enzymes’ types are known to be associated with several single-nucleotide polymorphisms (SNPs) [7]. Among these, the functional SNPs of *ALDH2* (*rs671*) have been associated with the development of cancers of the upper aerodigestive tract, head, and neck [7,8,9,10,11]. Individuals with the *ALDH2* polymorphism *rs671*, which reduces enzyme metabolic activity by 60–90%, may experience more exposure to AA, a known carcinogen [12]. This polymorphism is prevalent among East Asian populations, with approximately half of all Japanese individuals found to have an *ALDH2* deficiency phenotype, whereas it is almost entirely absent in other populations [7,13]. A recent study on *ALDH2* polymorphisms and the attributable burden of cancer in East Asia revealed that the estimated burden of alcohol-related cancer increased when *ALDH2* polymorphisms were considered. Notably, the highest population-attributable fraction of alcohol-related cancer in East Asia were observed for esophageal cancer, estimated at approximately 22–37% (China: 21–35%; Japan: 28–51%; South Korea: 40–55%) [14].

Somatic mutations are major contributors to various human cancers [15]. Whole-genome and exome sequencing analyses of human cancers have identified characteristic mutational profiles in a trinucleotide context, referred to as mutational signatures, across different cancer types [16,17]. These patterns are considered to result from an imbalance between DNA damage caused by exogenous and/or endogenous factors and the efficiency of the DNA repair system [18]. SBS16 is one such signature frequently observed in East Asia, including in esophageal, liver, and stomach cancers in the Japanese population [19,20,21,22]. Therefore, SBS16 is considered important in Japanese cancer development. Since SBS16 has been reported to be associated with the *ALDH2* polymorphism (*rs671*) and alcohol consumption, its etiology is thought to be linked to alcohol metabolism and its byproduct, AA [23,24]. Moreover, SBS16 predominantly exhibits T:A to C:G mutations with a strong transcribed strand bias. Strand bias occurs when DNA damage, such as adduct formation on the transcribed strand, is efficiently repaired by transcription-coupled repair (TCR), but remains unrepaired on the nontranscribed strand [25]. For SBS16, a strong strand bias occurred in the ATA and ATT contexts on the transcribed strand. Based on TCR activity, adenine adducts may contribute to the A to G (complementary from T to C) transition in this signature (COSMIC database; https://cancer.sanger.ac.uk/signatures/sbs/sbs16/, accessed on 11 June 2025). To date, only guanine adducts, such as *α*-S- and *α*-R-methyl-*γ*-hydroxy-1, *N*^2^-propano-2′-deoxyguanosine (CrPdG) and *N*^2^-ethylidenedeoxyguanosine (*N*^2^-ethylidene-dG), have been identified as alcohol-related adducts [26,27]. Notably, *N*^2^-ethylidene-dG is relatively unstable; however, it can be stabilized via chemical reduction to *N*^2^-ethyldeoxyguanosine (*N*^2^-ethyl-dG) in the presence of the reducing agent NaBH_3_CN. This stable product has been detected in the livers and esophagi of mice, with significantly increased levels observed in *Aldh2*-knockout mice after ethanol ingestion [28,29,30]. However, these guanine adducts cannot explain the relationship between AA and SBS16. Recently, a mass spectrometry-based DNA adductome approach was used to screen for additional alcohol-related DNA adducts [31]. A total of 22 DNA adducts were detected, including two novel adducts, *N*^6^-ethyldeoxyadenosine (*N*^6^-ethyl-dA) and *N*^4^-ethyldeoxycytidine, which were identified through comparison with synthesized standards. Furthermore, these adducts were detected in oral cells collected from volunteers exposed to alcohol, with a significant increase in the total observed after alcohol exposure. Considering the mutation pattern of SBS16 in an integrated manner, it can be inferred that *N*^6^-ethylidene-dA (*N*^6^-ethyl-dA) may serve as the driver adduct directly involved in SBS16. However, no information is available regarding the mutagenicity of *N*^6^-ethylidene-dA (*N*^6^-ethyl-dA). Moreover, the detailed mechanisms underlying alcohol-induced human cancer development and its direct association with SBS16 remain largely unresolved. As mentioned earlier, the *N*^2^-ethylidene adduct is relatively unstable, suggesting that it is non-mutagenic. Additionally, the possibility that the *N*^2^-ethylidene adduct may convert to the *N*^2^-ethyl adduct in vivo cannot be ruled out. Therefore, in this study, we investigated the mutagenic activity of *N*^6^-ethyl-dA.

To confirm the mutagenic potential of *N*^6^-ethyl-dA, we synthesized oligonucleotides containing the adduct and evaluated them using a primer extension assay with human DNA polymerase and the *supF* gene mutation test. However, *N*^6^-ethyl-dA lacked mutagenic potency in both the assays. Consequently, we sought to identify another candidate as the driver adduct of SBS16 via the DNA adductome approach. This approach is a comprehensive method for analyzing DNA adducts using liquid chromatography (LC)-high-resolution accurate mass spectrometry (HRAM-MS) [32,33,34]. HRAM-MS enables the acquisition of spectral data with precise mass measurements, allowing for the determination of the molecular formula of an ion. Additionally, the MS/MS fragmentation data can be used to detect DNA adducts and provide structural information or confirmation. Thus, the HRAM adductome approach is suitable for targeted and untargeted analyses [35]. Previously, we employed this approach to investigate the mutagenic mechanisms of nanosized magnetite in mouse lungs and the effects of 1,4-dioxane exposure on rat livers [32,34]. Furthermore, we used it to identify environmental factors contributing to the high incidence of esophageal cancer in Cixian, China [36], and to elucidate the molecular mechanisms underlying occupational bladder cancer related to *ortho*-toluidine exposure [37]. Through this approach, we identified an adenine adduct that was more abundantly produced in the reaction mixture of calf thymus DNA and AA than *N*^6^-ethyl-dA. After isolating and purifying this novel dA adduct, followed by MS and NMR spectral analyses, we determined its chemical structure as 3-(2′-deoxyribos-1′-yl)-7,9-dimethyl-3,9-dihydro-7*H*-[1,3,5]oxadiazino[4,3-*i*]purine (*N*^1^-oxydiethylidene-dA), in which two AA molecules bind to an adenine base. Additionally, we examined the in vivo formation of this novel DNA adduct.

## 2. Materials and Methods

### 2.1. Chemicals

2′-Deoxyadenosine, bovine spleen phosphodiesterase II, and high-performance liquid chromatography (HPLC)-grade acetonitrile were purchased from Wako Pure Chemical Industries, Ltd. (Chuo-ku, Osaka, Japan). AA, calf thymus DNA, DNase II from porcine spleen, and acid phosphatase from potato were obtained from Sigma-Aldrich Co. LLC (St. Louis, MO, USA). DNA Hydration Solution was sourced from QIAGEN (Valencia, CA, USA). *N*^6^-ethyl-dA and its phosphoramidite were purchased from Granlen Inc. (Zhengzhou, China). All other chemicals were of analytical grade and were acquired from Wako Pure Chemical Industries, Ltd.

### 2.2. Primer Extension Assay

Human pol α and pol η were procured from CHMERX (Milwaukee, WI, USA) and Enzymax (Lexington, KY, USA), respectively. Human pol κ was purified as previously described [38]. 5′-Cy3 oligonucleotides used for primers and unmodified/*N*^6^-ethyl-dA-modified template oligonucleotides were purchased from Hokkaido System Sciences Co., Ltd. (Sapporo, Hokkaido, Japan). Primer extension reactions for pols α, κ, and η were conducted at 25 °C in a reaction buffer (10 µL) containing 40 mM Tris-HCl (pH 8.0), 60 mM KCl, 5 mM MgCl_2_, 10 mM dithiothreitol, 250 μg/ml bovine serum albumin, and 2.5% glycerol. To assess translesion DNA synthesis past *N*^6^-ethyl-dA, the reactions included four deoxyribonucleoside triphosphates (dNTPs) (100 µM each) and an unmodified or *N*^6^-ethyl-dA-modified 38-mer DNA template (750 fmol, 5′-CATGCTGATGAATTCCTTCXCTTCTTTCCTCTCCCTTT, where X represents dA or *N*^6^-ethyl-dA), primed with a Cy3-labeled 10-mer primer (500 fmol, 5′-AGAGGAAAGA). For single-nucleotide insertion reactions, the reaction buffer contained 100 µM dATP, dCTP, dGTP, or dTTP and an unmodified or *N*^6^-ethyl-dA-modified 38-mer DNA template (750 fmol) primed with a Cy3-labeled 12-mer primer (500 fmol, 5′-AGAGGAAAGAAG). The reactions were initiated by adding varying amounts of pols and terminated by adding 2 µL of formamide dye containing 100 mg/mL blue dextran and 50 mM EDTA. The reaction products were separated via 20% polyacrylamide gel electrophoresis and visualized using the iBright Imaging System (Thermo Fisher Scientific, Waltham, MA, USA).

### 2.3. supF Forward Mutation Assay

Position 120 of the *supF* gene was selected as the site for *N*^6^-ethyl-dA modification. The 5′-phosphorylated 21-mer primer (5′-CGACTTCGANGGTTCGAATCC-3′, where N represents dA or *N*^6^-ethyl-dA) was obtained from Hokkaido System Sciences Co., Ltd. (Hokkaido, Japan). The synthesized primer was annealed with pMY189 single-stranded DNA, which was prepared using the VCSM13 helper phage. The double-stranded plasmid DNA was then synthesized and purified via ultracentrifugation as previously described [39].

For the mutation assay, the human lymphoblastoid TK6 cell line was cultured in RPMI-1640 medium (Nacalai Tesque, Kyoto, Japan) supplemented with 200 μg/mL sodium pyruvate, 100 U/mL penicillin, 100 μg/mL streptomycin, and 10% (*v*/*v*) heat-inactivated horse serum (Thermo Fisher Scientific). The cells were maintained at 37 °C in an atmosphere of 5% CO_2_ and 100% humidity. The synthesized plasmid DNA (500 ng) was introduced into 2 × 10^6^ cells using the NEPA21 Super Electroporator (Nepa Gene Co., Ltd., Ichikawa-shi, Chiba, Japan) following the manufacturer’s recommendations. After 48 h of incubation, the replicated plasmid was extracted from the cells and digested with *Dpn*I to remove unreplicated plasmids. The resulting plasmid was then introduced into the *Escherichia coli* KS40/pOF105 indicator strain. To select *E. coli* with a mutated *supF* gene, the transformed cells were plated on LB selection plates containing nalidixic acid (50 μg/mL), streptomycin (100 μg/mL), ampicillin (150 µg/mL), chloramphenicol (30 μg/mL), X-Gal (80 μg/mL), and IPTG (23.8 μg/mL). To determine the total number of transformants, the cells were plated onto LB titer plates containing ampicillin (150 μg/mL) and chloramphenicol (30 μg/mL). The *supF* mutant frequency was calculated by dividing the number of colonies on selection plates by the number of colonies on titer plates [39].

### 2.4. Reaction of AA and Calf Thymus DNA

Calf thymus DNA (CT-DNA) was dissolved in DNA Hydration Solution (QIAGEN, Hilden, Germany) to a final concentration of 2 mg/mL. A 1.5 mL aliquot of this solution and 750 μL of AA were added to a 2 mL Eppendorf tube, vortexed, and incubated at 37 °C for 24 h. Due to the volatility of AA, the reaction was performed in excess volume to prevent evaporation gaps in the 2 mL reaction tube. A control sample was prepared by adding distilled water instead of AA. DNA was isolated using ethanol precipitation after adding NaBH_3_CN at a concentration of 100 mM and stored at −80 °C until further use.

### 2.5. DNA Adductome Analysis

The DNA samples were enzymatically digested as previously reported [36]. LC-HRAM analyses were performed using a Shimadzu Prominence LC system (Kyoto, Japan) interfaced with a Triple TOF6600 mass spectrometer (SCIEX, Framingham, MA, USA) in Information-Dependent Acquisition Scanning mode. The HPLC conditions were as follows: column = Synergi^TM^ Fusion-RP (2.5 μm particle size, 2.0 × 100 mm; Phenomenex, Torrance, CA, USA); flow rate = 0.4 mL/min; and solvent system = a linear gradient from 2.5% to 85% acetonitrile in 10 mM ammonium acetate (pH 5.3) over 30 min controlled by Analyst TF 1.7.1 software. The sample injection volumes were 10 μL each. The MS parameters were as follows: mass range scanned from 50 to 1000 *m*/*z* with a scan duration of 0.5 s (1.0 s total duty cycle), capillary voltage of 3.7 kV, sampling cone voltage of 40 V, extraction cone voltage of 4 V, source temperature of 125 °C, and desolvation temperature of 250 °C. Nitrogen gas was used as the desolvation gas (flow rate: 800 L/h) and cone gas (30 L/h). All the data were collected in positive ion mode, with a cone voltage of 20 V and collision energy of 5 V.

**Figure 1:**
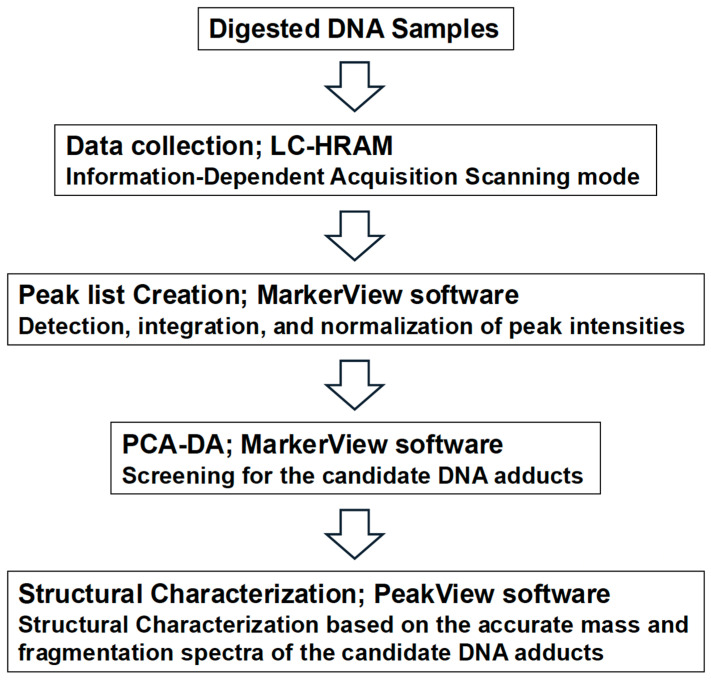
DNA adductome workflow for discovery of candidate DNA adducts.

Figure 1 shows the adductome analysis workflow screening for the candidate DNA adducts. The raw data files obtained from the LC-HRAM runs were analyzed using PeakView^®^ 2.1 and MarkerView™ 1.3 software (SCIEX). These applications detect, integrate, and normalize peak intensities relative to the total peak intensity within each sample. The resulting multivariate dataset, which included the peak number (based on retention time and *m*/*z*), sample name, and normalized peak intensity, was analyzed using principal component analysis–discriminant analysis (PCA–DA).

### 2.6. Structural Analysis of Novel AA-dA Adducts

AA was incubated with 2′-dA in DNA Hydration Solution at 37 °C for 24 h, and the resulting solution was separated by HPLC (SHIMADZU UFLCXR DGU-20A5 LC-20AD, Shimadzu Co. Jp., Nakagyou-ku, Kyoto, Japan). The HPLC conditions were as follows: column = InertSustain C18 (5 μm particle size, 4.6 × 250 mm; GL Science, Tokyo, Japan); flow rate = 0.8 mL/min; and solvent system = isocratic 5% acetonitrile in 0.1 M ammonium acetate (pH 5.3) for 0–10 min, followed by a linear gradient from 5% to 80% acetonitrile in 0.1 M ammonium acetate over 30 min. The column oven was set at 40 °C. In addition to a few peaks detected in the solution without 2′-dA, several new peaks with retention times between 19 and 23 min were observed in the reaction mixture with AA and 2′-dA. These peaks were collected and analyzed by LC-HRAM to confirm their *m*/*z* values and MS/MS fragment data. All the data were collected with collision energies of 15 V. The collected samples were then subjected to ^1^H-NMR spectral analysis.

^1^H-NMR (400 Mhz, DMSO-*d*_6_): δ 8.46 (1H), 8.30 (1H), 5.72 (1H), 5.12 (1H), 1.78 (3H), 1.50 (3H). The chemical structure and proton signal assignments were determined using DQF-COSY, HMQC, and HMBC spectra (Figure S2).

### 2.7. Analysis of AA-DNA Adducts in the Liver of Mice Treated with Alcohol

A total of 21 male C57BL/6J mice purchased from Charles River Japan, Inc. (Atsugi, Japan) were provided with food (AIN-76A powder diet, CLEA Japan Inc, Meguro-ku, Tokyo, Japan) and tap water ad libitum. After one week of acclimatization, the animals, at 7 weeks of age, were divided into three groups—Group 1 (control, n = 5): standard diet and distilled water; Group 2 (n = 8): 10% ethanol in drinking (distilled) water; and Group 3 (n = 8): 10% ethanol in drinking (distilled) water and 0.04% disulfiram (Wako Pure Chemical Industries, Ltd.,Chuo-ku, Osaka, Japan Chuo-ku, Osaka, Japan) in the diet after four weeks of rearing. The mice were subsequently euthanized. The livers were excised and stored at −80 °C until DNA extraction. All the animal experiments were conducted in accordance with protocols approved by the Committee for the Ethics of Animal Experimentation of the National Cancer Center (approval protocol no. T17-029-M05, 10 September 2020). DNA from liver tissues was extracted and purified using the Gentra^®^ Puregene™ Tissue Kit (QIAGEN, Hilden, Germany) following the manufacturer’s instructions, except that NaBH_3_CN was added to all solutions. The extracted DNA was stored at −80 °C until DNA adduct analysis. The extracted DNA (100 μg) was digested with DNaseI, nuclease P1, and alkaline phosphatase as previously described [36]. The amounts of *N*^6^-ethyl-dA and the novel AA-dA were measured by an LC-30 CE high-performance liquid chromatograph with an SIL-30 AC sampler and a CTO-20 AC column oven interfaced with an LC-MS-8045 triple quadrupole mass spectroscopy system (Shimadzu Co., Kyoto, Japan). The analysis of processed samples was performed with the electrospray ionization (ESI) source in positive ion mode using multiple reaction monitoring (MRM). The HPLC conditions were as follows: column = Inertsustain C18 (5 μm particle size, 4.6 mm × 250 mm; GL Sciences, Tokyo, Japan); flow rate = 0.8 mL/min; the column temperature was set to 40 °C; and 10 mM ammonium acetate (pH 5.3) and acetonitrile were used as mobile phases A and B, respectively. Chromatographic separation was performed by gradient elution: 0–10 min, 5% B; 10–30 min, and linear gradient from 5% to 80% B. The instrument settings for the mass spectrometer were set as follows: nebulizing gas flow, 8 L/min; drying gas flow, 10 L/min; heating gas flow, 10 L/min; interface temperature, 400 °C; desolvation line temperature, 250 °C; and heat block temperature, 400 °C. The major fragment ions detected were *m*/*z* 251.1→136.1 (2′-dA), *m*/*z* 280.1→164.1 (*N*^6^-ethyl-dA) and *m*/*z* 322.1→206.1 (*N*^1^-oxydiethylidene-dA), corresponding to the loss of the deoxyribose (dR) moiety. Quantification was performed using a standard curve for *N*^6^-ethyl-dA or 2′-dA (*N*^1^-oxydiethylidene-dA).

## 3. Results

### 3.1. Mutagenic Activity of N^6^-Ethyl-dA Adducts

**Figure 2:**
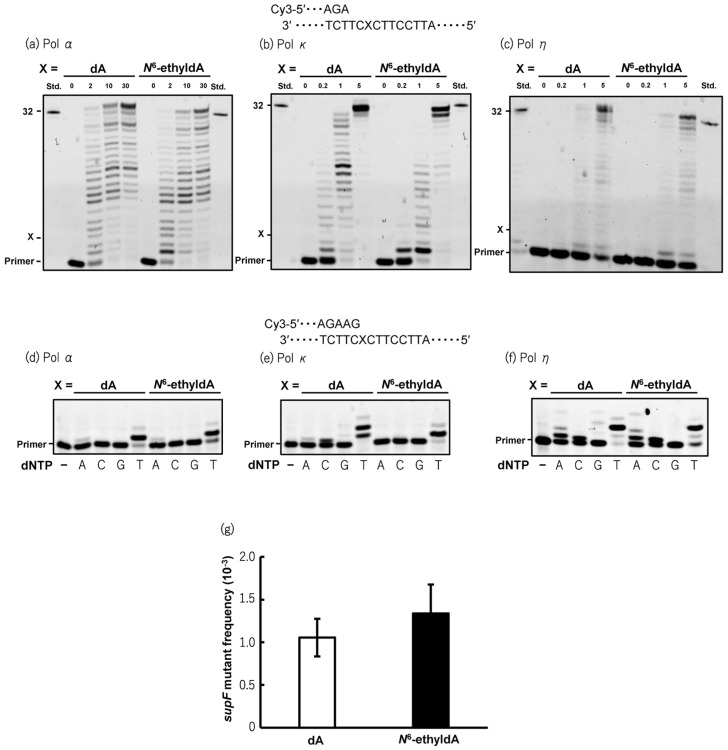
Mutagenic potential of *N*^6^-ethyl-dA in vitro and in cellulo. Primer extension reactions catalyzed by human pol α (**a**), pol κ (**b**), and pol η (**c**) were performed using unmodified or *N*^6^-ethyl-dA-modified 38-mer template DNA annealed to Cy3-labeled 10-mer primer. X indicates position of dA or *N*^6^-ethyldA on template strand. Reactions were initiated by addition of varying amounts of pol α (2, 10, or 30 nM), pol κ (0.2, 1, or 5 nM), and pol η (0.2, 1, or 5 nM), followed by incubation at 25 °C for 30 min in presence of four dNTPs. Reaction mixtures were subjected to 20% denaturing PAGE, where shifted bands indicate extended products generated by pols. Thirty-two-mer standard marker (std) for fully extended products was loaded in lanes on both sides of gels. Incorporation of deoxyribonucleoside monophosphates (dNMPs) opposite dA or *N*^6^-ethyl-dA catalyzed by human pol α (**d**), pol κ (**e**), and pol η (**f**) was assessed using unmodified or *N*^6^-ethyl-dA-modified 38-mer template DNA annealed to Cy3-labeled 12-mer primer. Reactions were carried out at 25 °C for 10 min in reaction buffer containing single dNTP (A; dATP, C; dCTP, G; dGTP, or T; dTTP) (100 μM) with pol α (10 nM), pol κ (5 nM), or pol η (5 nM). Extended products were separated by 20% denaturing PAGE. Shifted bands indicate incorporation of dNMP opposite dA or *N*^6^-ethyldA. First lanes (−) represent control results without dNTP in reaction mixture. (**g**) *supF* mutant frequencies in unmodified or *N*^6^-ethyl-dA-modified plasmids propagated in TK6 cells. Transfection experiments were performed four times. Data are expressed as mean ± standard error. Original western blots can be found at Supplementary Materials.

To investigate the mutagenic activity of *N*^6^-ethyl-dA, an oligonucleotide containing a single adduct was chemically synthesized. To assess the effect of *N*^6^-ethyl-dA on DNA synthesis by human DNA polymerases, in vitro primer extension reactions were conducted using the *N*^6^-ethyl-dA-modified 38-mer DNA templates in the presence of four NTPs and recombinant human Pols α, κ, and η. Although primer extension catalyzed by Pol κ was slightly hindered by the presence of *N*^6^-ethyl-dA, Pols α and η efficiently bypassed *N*^6^-ethyl-dA at a rate comparable to that of unmodified dA (Figure 2a–c). During lesion bypassing, Pols α, κ, and η incorporated dTMP opposite *N*^6^-ethyl-dA, consistent with reactions using the unmodified template (Figure 2d–f). To further evaluate whether *N*^6^-ethyl-dA exhibited mutagenic potential in human cells, an *supF* mutation assay was performed using a plasmid containing a single *N*^6^-ethyl-dA propagated in human TK6 cells. The *supF* mutant frequency induced by *N*^6^-ethyl-dA (1.3 ± 0.34 × 10^−3^) was not significantly different from that of the control plasmid (1.1 ± 0.22 × 10^−3^) (Figure 2g), suggesting that *N*^6^-ethyl-dA is a non-mutagenic DNA lesion in human cells.

### 3.2. Comprehensive Analysis of AA-DNA Adducts

#### 3.2.1. Comprehensive Analysis of DNA Adducts Induced by AA Treatment

**Figure 3:**
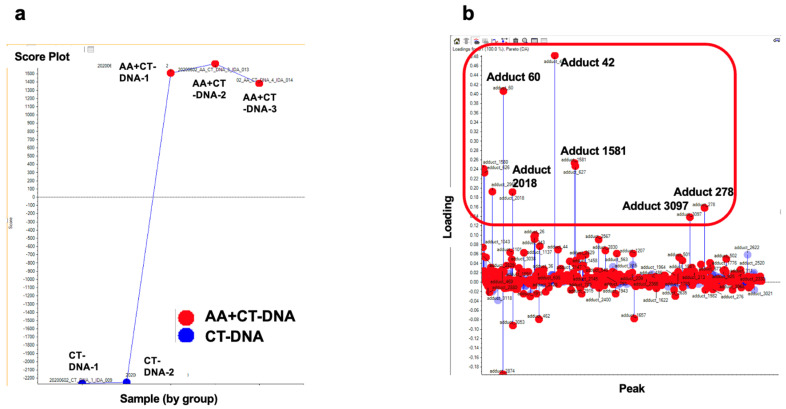
Comprehensive DNA adduct analysis. (**a**) Two-dimensional PCA–DA scores of DNA adducts obtained from adductome analysis. Blue: Control (CT-DNA); red: AA-treated CT-DNA. (**b**) Variable loading plots. Each red spot represents DNA adducts observed in DNA adductome analysis. Adducts in upper area located away from baseline are characteristic of AA-treated group, and adducts at bottom located away from baseline are characterisDNA adducts enclosed within the red-square region indicate candidate characteristic DNA adducts for AA exposuredducts for AA exposure.

Since *N*^6^-ethyl-dA exhibits no mutagenic activity, we sought to identify another potential driver adduct of AA that could account for SBS16. When the DNA samples from the in vitro reactions were subjected to DNA adductome analysis, the two-dimensional (2D) PCA–DA scores plot of DNA adducts show a distinct clustering of control- and AA-treated DNA. The associated loadings plot indicates that several DNA adducts contributed significantly to AA treatment based on their PCA significance (Figure 3).

#### 3.2.2. Screening for the Novel DNA Adducts Related to AA Exposure

**Figure 4:**
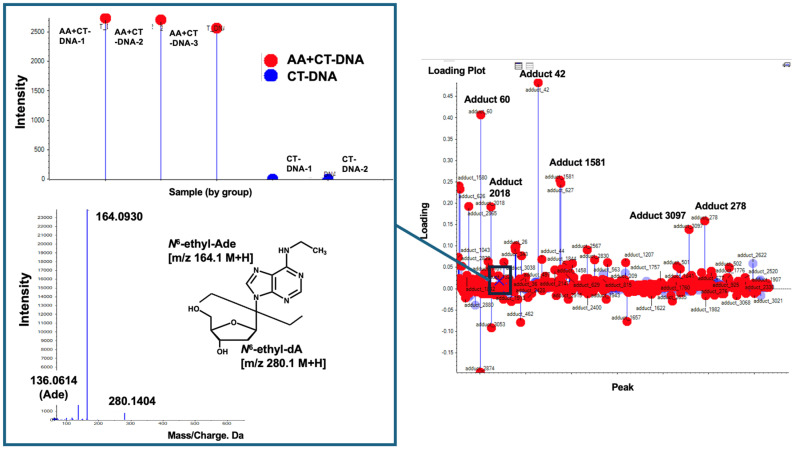
Search for *N*^6^-ethyl-dA. *N*^6^-ethyl-dA was screened using its *m*/*z* values of 280.1→164.1 as indicator. *N*^6^-ethyl-dA, marked by “X”, was observed near baseline, suggesting that this DNA adduct does not significantly contribute to AA treatment. Square boxes indicate MS/MS fragment data and its intensity in each sample for this DNA adduct.

First, the previously reported dA adduct, *N*^6^-ethyl-dA, was screened using its *m*/*z* as an indicator. As a result, an ion peak at *m*/*z* 280.1, corresponding to *N*^6^-ethyl-dA, was detected, along with a fragment ion peak at *m*/*z* 164.1, corresponding to the loss of the -dR moiety, as observed in the MS/MS data. This confirmed the identification of the DNA adduct as *N*^6^-ethyl-dA (Figure 4). Its intensity was high in all the samples of AA-treated CT-DNA, whereas it was barely detected in the CT-DNA. However, the loading plot data showed that this DNA adduct appeared near the baseline, indicating that several other DNA adducts contribute more significantly to AA treatment.

**Figure 5:**
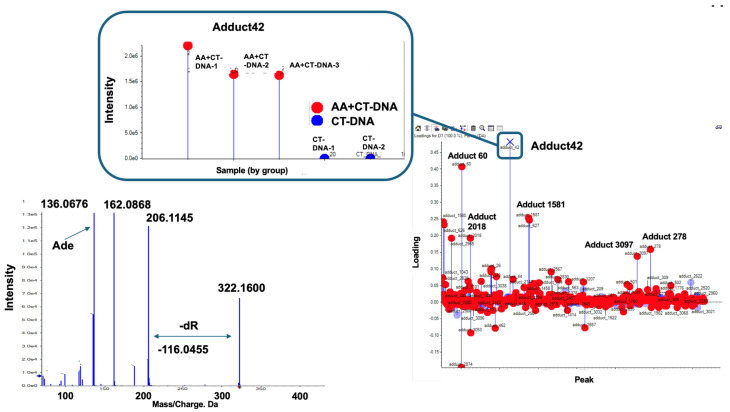
Screening for novel DNA adduct. In this loading plot, DNA Adduct 42 can be observed at furthest distance from baseline, exhibiting higher intensity (1.5~2.0 × 10^6^) compared to that of *N*^6^-ethyl-dA in Figure 3. Adduct 42 is marked by “X”. Square box indicates intensity of each samples. Left panel shows MS/MS fragmentation pattern of Adduct 42. Fragment ion peak (*m*/*z* 206.11) corresponds to loss of dR (−116.04) moiety from precursor ion peak (*m*/*z* 322.16); in addition, ion peak corresponding to adenine (*m*/*z* 136.0676) can be observed. Thus, novel adduct of 2′-dA may be produced by AA treatment.

Figure 5 shows the DNA adducts that contribute most significantly to AA treatment. Among them, an adduct named Adduct 42 was observed at the greatest distance from the baseline, with a significantly higher intensity (1.5 − 2.0 × 10^6^) compared to those of the other candidate characteristic DNA adducts and *N*^6^-ethyl-dA in the AA treatment group. The MS/MS fragmentation pattern of this adduct revealed a fragment ion peak corresponding to the loss of a dR (−116.0467) moiety from the precursor ion peak (*m*/*z* 206.1145). Additionally, an ion peak corresponding to adenine (*m*/*z* 136.0676) was detected. These findings suggest that a novel adduct of 2′-dA may be produced by AA treatment.

Based on the MS/MS fragmentation data and the *m*/*z* values, additional DNA adducts contributing to AA exposure were identified as follows: Adduct 1581 [*m*/*z* 338.1460] for the DNA adducts containing a dG and AA moieties. Furthermore, the MS/MS fragmentation pattern of Adduct 278 included ion peaks corresponding to Adduct 42 [*m*/*z* 136.0619, 162.0781, 206.1132, 322.1524] and additional peaks [*m*/*z* 402.1213, 518.1770]. These additional peaks were suggested to represent Adduct 42 + PO_3_ [*m*/*z* 322.1524 + 79.9689 corresponding to PO_3_ = 402.1213] and Adduct 42 + PO_3_ + dR (*m*/*z* 322.1524 + 79.9689 + 116.0557, corresponding to dR = 518.1770). These results are shown in Figure S1. Given that we focused on the novel AA-related dA adduct, further analysis was conducted on Adduct 42. The other DNA adducts located in the upper area, identified as dA (Adduct 60), dG (Adduct 2018), and dT (Adduct 3097) based on their MS/MS fragmentation data (Figure S1), are potential contaminants because of the overflow of large amounts of normal nucleotides.

### 3.3. Structural Analysis of Novel AA-dA Adduct

**Figure 6:**
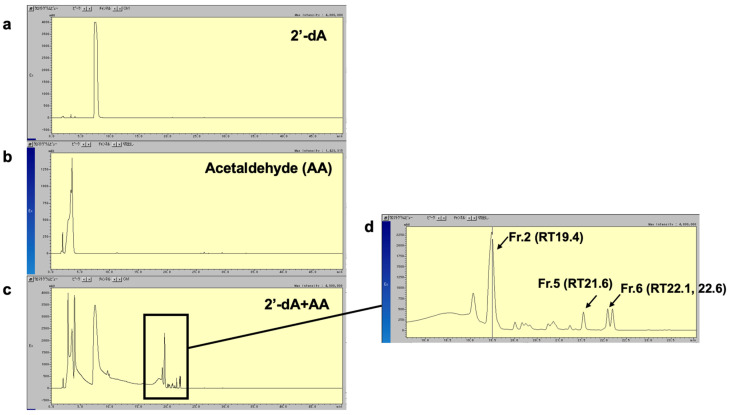
HPLC profile of AA-dA adducts. HPLC chromatogram of 2′-dA (**a**) and AA (**b**) and aliquot of reaction mixture of 2′-dA with AA (**c**). UV absorbance of eluate was monitored at 254 nm. Major peaks, corresponding to reaction products of 2′-dA and AA, are indicated by arrows. (**d**) Magnified view of the reaction products Fr.2, Fr.5, and Fr.6 of 2′-dA and AA.

To analyze the chemical structure of the newly identified AA-dA adduct, named Adduct 42, we attempted to react 2′-dA with AA in vitro and isolate the resulting reaction product. Figure 6 shows the HPLC patterns of 2′-dA (Figure 6a) and AA (Figure 6b) and the reaction mixture of 2′-dA and AA (Figure 6c). The chromatogram patterns of the reaction mixture (Figure 6c) revealed several peaks, which were presumed to be reaction products of 2′-dA and AA. A magnified view is shown in Figure 6d, in which we isolated and purified Fr.2, Fr.5, and Fr.6, the major peaks, for further chemical structural analysis. Fr.2 and Fr.5 were identified as singlet peaks, whereas Fr.6 appeared as a doublet peak, suggesting that this substance might be a structural isomer.

**Figure 7:**
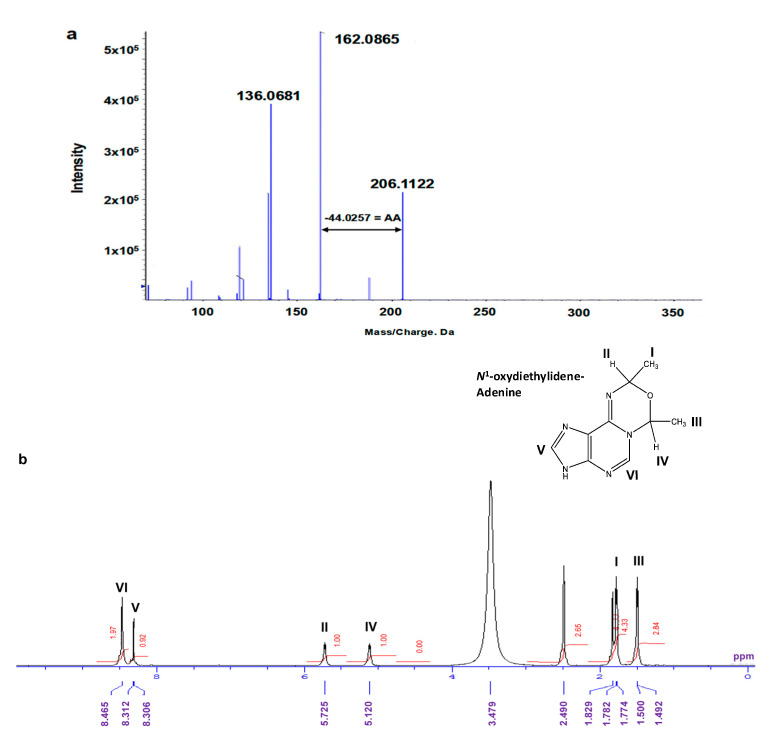
Chemical structural analysis of isolated compound in Fraction 2. (**a**) MS/MS fragmentation pattern. Fragment ion peak (*m*/*z* 162.08) corresponding to loss of AA (−44.02) moiety from precursor ion peak (*m*/*z* 206.11); in addition, ion peak corresponding to adenine (*m*/*z* 136.06) was observed. (**b**) ^1^H-NMR spectra of isolated compound in Fraction 2. ^1^H-NMR (400 Mhz, DMSO-*d*6): δ 8.46 (1H), 8.30 (1H), 5.72 (1H), 5.12 (1H), 1.78 (3H), and 1.50 (3H). Proton at 5.1 ppm was correlated with proton at 1.5 ppm, as determined through DQF-COSY experiments. Additionally, methine proton at 5.7 ppm was correlated with protons at 1.7 ppm. Methyl protons at 1.7 ppm further correlated with C2 position, as determined via HMBC proton–carbon correlation spectroscopy (Figure S2).

By repeating the HPLC procedure, we were able to collect approximately 1000 μg of Fr.2 for ^1^H-NMR spectral analysis. However, due to the low yields, we were unable to collect a sufficient amount of Fr.5 and Fr.6 for further analysis. Figure 7a shows the MS/MS fragmentation pattern of Fr.2, revealing a precursor ion peak at *m*/*z* 206.1122 and a daughter ion peak at *m*/*z* 162.0865, with a neutral loss of 44.0257, corresponding to the AA moiety. Moreover, an ion peak corresponding to adenine (*m*/*z* 136.0681) was also detected. Figure 7b presents the ^1^H-NMR spectrum of Fr.2 measured in DMSO-*d*_6_. From ^1^H-NMR spectroscopy, we observed two methyl proton signals at 1.5 ppm (3 protons) and 1.7 ppm (3 protons), as well as two methine protons at 5.1 and 5.7 ppm, respectively. The proton at 5.1 ppm was correlated with the methyl protons at 1.5 ppm in the DQF-COSY experiments, indicating their close proximity. Similarly, the methine proton at 5.7 ppm was correlated with the protons at 1.7 ppm. Protons in the aromatic region appeared at 8.3 and 8.4 ppm as singlets, corresponding to the adenine structures at the positions C8 and C2, respectively. The methyl protons at 1.7 ppm were correlated with carbon at the C2 position, as determined via HMBC proton–carbon correlation spectroscopy (Figure S2). By combining the NMR carbon–proton correlation data with the MS fragmentation patterns, we determined the compound’s chemical structure is 7,9-dimethyl-3,9-dihydro-7*H*-[1,3,5]oxadiazino[4,3-*i*]purine (*N*^1^-oxydiethylidene-adenine) (Figure 7b).

**Figure 8:**
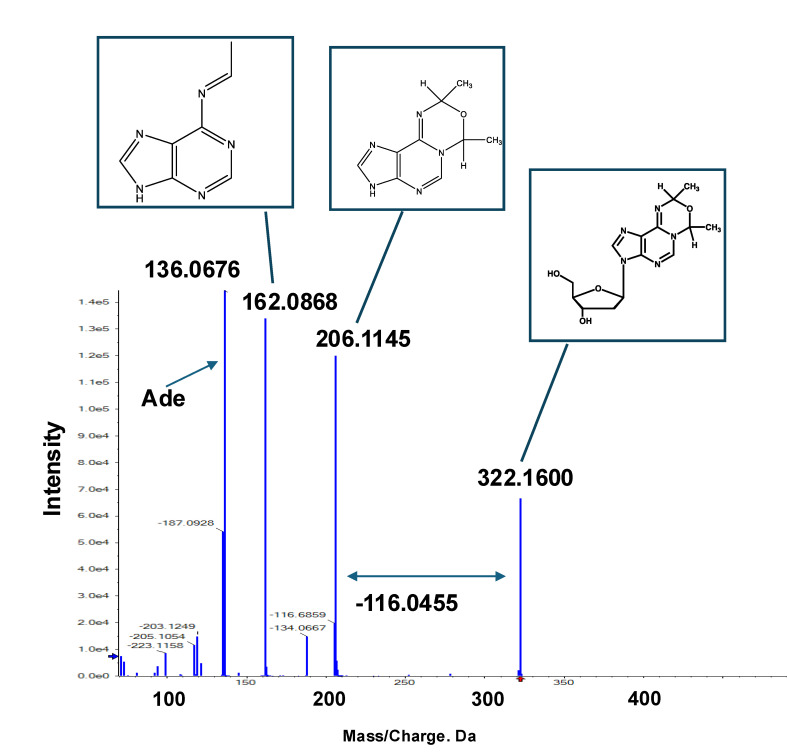
Tandem mass spectrometry (MS/MS) fragmentation pattern and corresponding chemical structures of Adduct 42. Based on NMR spectral analysis of Fr.2 and commonly observed fragment ion peaks in Fr.6, chemical structure of Adduct 42 was identified as *N*^1^-oxydiethylidene-dA.

As mentioned above, Fr.6 exhibits a doublet peak, and its MS/MS fragmentation patterns are nearly identical, indicating that it is a structural isomer (Figure S3). As the precursor and daughter ion peaks at *m*/*z* 366.17 and 322.15 corresponded to a neutral loss of 44.026, which is associated with the AA moiety, the chemical structure of Fr.6 must contain at least one AA moiety. Additionally, a fragment ion peak at *m*/*z* 250.13, corresponding to the loss of a dR moiety (−116.04) from the precursor ion peak (*m*/*z* 366.17), was observed. Another loss of a dR moiety (−116.04) from the daughter ion peak (*m*/*z* 322.15) resulted in a fragment ion at m/z 206.10. Based on NMR structural analysis, we identified *m*/*z* 206.10 as *N*^1^-oxydiethylidene-adenine, whereas *m*/*z* 162.07 represents its degradation product. These fragment ion peaks (*m*/*z* 206.10 and 162.07) were observed in all the fractions, including Fr.2, 5, and 6, as well as in Adduct 42 (Figure 5, Figure 7a, and Figure S3), indicating that these compounds share a partial *N*^1^-oxydiethylidene-adenine moiety. Given the neutral loss of −116.04 (corresponding to the dR moiety) observed between *m*/*z* 322.1 and 206.1, we identified the *m*/*z* 322.15 ion peak in Fr.6 and Adduct 42 as 3-(2′-deoxyribos-1′-yl)-7,9-dimethyl-3,9-dihydro-7*H*-[1,3,5]oxadiazino[4,3-*i*]purine (*N*^1^-oxydiethylidene-dA) (Figure 8). Furthermore, because a neutral loss of −44.026 (corresponding to the AA moiety) was observed between *m*/*z* 366.17 and 322.15, the ion peak at *m*/*z* 366.17 in Fr.6 was presumed to be an exocyclic-dA adduct containing three AA moieties.

### 3.4. Formation of Novel AA-dA Adducts in the Livers of Mice After Administration of Ethanol

To confirm the formation of a novel AA-related DNA adduct in vivo, ethanol was administered at a concentration of 10% in drinking water with or without the ALDH2 inhibitor disulfiram for four weeks to male mice. During the experimental period, water intake dropped to 80% of the control group in the 10% ethanol group, whereas food intake remained similar to that of the control group. No weight suppression was observed in ethanol-treated groups, suggesting no toxicity under these conditions. The levels of *N*^1^-oxydiethylidene-dA and *N*^6^-ethyl-dA in the liver were analyzed using LC-ESI-MS in MRM mode. A peak at *m*/*z* 322.1→206.1, corresponding to *N*^1^-oxydiethylidene-dA, was detected in all the groups. The baseline adduct level was almost comparable to that of CrPdG, having a similar chemical structure to *N*^1^-oxydiethylidene-dA, as reported in the previous report [29]. The LC-MS chromatograms are shown in Figure S4. The adduct levels were elevated in the ethanol-treated group and were further increased in the group treated with the ALHD2 inhibitor disulfiram (Table 1). However, another AA-related-dA adduct, *N*^6^-ethyl-dA, was also detected in the liver samples, but its levels were lower than those of *N*^1^-oxydiethylidene-dA.

## 4. Discussion

Alcohol consumption is a causative factor in human cancer development, particularly emphasized in the well-investigated relationship between *ALDH2* polymorphism, *rs671*, and esophageal cancer risk. The carcinogenic mechanism of alcohol is primarily attributed to the in vivo formation of the carcinogen AA as its metabolite [2,3,26]. The relationship between DNA adduct formation, mutagenesis, and carcinogenesis is generally well understood and accepted. It has already been reported that AA forms several DNA adducts [26,27,40]. AA reacts with the exocyclic amino group of dG to form *N*^2^-ethylidenedeoxyguanosine (*N*^2^-ethylidene-dG), recognized as the major DNA adduct of AA [26,40,41,42]. However, this adduct is relatively unstable and can be stabilized by chemical reduction with NaBH_3_CN to form the stable product *N*^2^-ethyldeoxyguanosine (*N*^2^-ethyl-dG) [43]. Due to the instability of *N*^2^-ethylidene-dG, no direct information on its mutagenicity is available, but it is believed to be non-mutagenic, as it would be lost from DNA before introducing misincorporation at the opposite lesion. However, the possibility that *N*^2^-ethylidene-dG is converted to *N*^2^-ethyl-dG in vivo cannot be ruled out, though its genotoxicity would still be low. Some reports indicate that human DNA polymerase can bypass the adduct and correctly insert CMP opposite the lesion [44,45,46,47]. Based on these findings, *N*^2^-ethyl-dG (i.e., *N*^2^-ethylidene-dG) is suggested as an excellent biomarker of AA exposure. Other AA-related DNA adducts have also been identified, such as those formed when two AA molecules react with 2′-dG, producing α-S- and α-R-methyl-γ-hydroxy-1, *N*^2^-propano-2′-deoxyguanosine (CrPdG) [26,27]. Unlike *N*^2^-ethyl-dG, CrPdG induces mutations, predominantly from G to T/A, followed by from G to C in human cells via translesion DNA synthesis [48]. Since AA induces G to A transitions in the *TP53* and *HPRT* genes in human-derived mammalian cells [49,50], CrPdG may contribute to these mutations. In addition to single-base substitutions, AA also induces specific tandem-base substitutions, such as from GG to TT, in double-stranded shuttle vectors treated with AA [51]. These tandem substitutions may result from DNA–DNA crosslinking. CrPdG adducts exist in two forms: ring-opened and ring-closed [27]. In its aldehyde ring-opened form (*N*^2^-(3-oxopropyl)-dG aldehyde), this adduct reacts with deoxyguanosine on the opposite strand, forming an interstrand crosslink [52]. However, this interstrand crosslink cannot fully explain the specific GG to TT base substitutions associated with AA exposure, suggesting the presence of an intrastrand crosslink. Supporting this hypothesis, a novel GG intrastrand crosslink has been observed in AA-exposed synthetic oligomers [53]. While interstrand crosslinks are attributed to CrPdG, the authors propose that *N*^2^-ethyl-dG is the basis for GG intrastrand crosslink formation. Initially, *N*^2^-ethyl-dG forms on one guanine base, which then binds to the exocyclic amino group of a neighboring guanine, creating an intrastrand crosslink. Additionally, an ethenobase adduct has been identified as a secondary AA-related DNA adduct, resulting from AA-induced lipid peroxidation in vivo [54]. Exposure to AA has been shown to increase the 1, *N*^2^-etheno-dG (NεdG) levels in mammalian cells [28]. While NεdG exhibits mutagenic activity, its mutation spectrum is complex, involving multiple base deletions and rearrangements near the adducted site [55]. Beyond the guanine base adducts, *N*^6^-ethyl-dA and *N*^4^-ethyldeoxycytidine have been identified using an adductome approach [31], though their mutagenic potential remains unclear. As outlined above, substantial information is available on the AA-related DNA adducts and their mutagenicity, which may help elucidate the link between alcohol consumption and cancer development. However, proving a direct causal relationship between specific DNA adducts and particular cancer types remains challenging, and despite these insights, the detailed carcinogenic mechanism of alcohol remains unresolved.

However, several mutation signatures have recently been reported, in which the somatic mutation data obtained from whole-genome/exome analyses using next-generation sequencing are classified into 96 types. Some of these mutation signatures have been used to establish links between environmental factors and human carcinogenesis. To date, approximately 100 signatures have been registered in the COSMIC database [56], among which SBS16 is frequently observed in esophageal cancer in Japanese patients [19]. Furthermore, SBS16 has been associated with *ALDH2* polymorphism (*rs671*) and alcohol consumption, with its characteristic T:A to C:G transition thought to be induced by alcohol (acetaldehyde) exposure [23,24]. As described above, although extensive information is available on the AA-related DNA adducts and their mutational patterns, the specific DNA adducts responsible for SBS16-specific mutations (T:A to C:G transition) have not yet been elucidated. In this study, the mutagenicity of *N*^6^-ethyl-dA, a known AA-related DNA adduct potentially responsible for SBS16, was evaluated using a primer extension assay and an *supF* gene mutation test with oligonucleotides containing synthesized *N*^6^-ethyl-dA. The findings confirmed that *N*^6^-ethyl-dA is not mutagenic using either assay. This result is consistent with the previous findings, as *N*^2^-ethyl-dG, which has a similar structure to *N*^6^-ethyl-dA, is also non-mutagenic.

To identify another candidate as the driver adduct of SBS16, we employed a DNA adductome approach and discovered a novel AA-dA adduct that appears structurally similar to CrPdG: the *N*^1^-oxydiethylidene-dA adduct with two AA molecules attached to an adenine base. The adduct level was significantly higher than that of *N*^6^-ethyl-dA in the reaction mixture of DNA and AA. Moreover, this adduct was actually produced in vivo, and its levels were markedly increased in the mouse livers following exposure to ethanol alone or in combination with disulfiram. The baseline adduct levels were almost comparable to those of CrPdG reported in a previous report [29]. While the *N*^1^-oxydiethylidene-dA levels were gradually increased with ethanol and ethanol plus disulfiram administration, the CrPdG levels were almost stable regardless of the ethanol treatment. Currently, we cannot provide an appropriate explanation for this discrepancy, but we might be able to determine the reaction activity between dA or dG and AA, i.e., the formation of *N*^1^-oxydiethylidene-dA is much more intense than that of *N*^2^-ethylidene-dA (*N*^2^-ethyl-dA) in the reaction of CT-DNA and AA in the present study. However, Guidolin et al. reported the presence of several alcohol-related DNA adducts, including *N*^6^-ethyl-dA, using an adductomics approach [31]. Among these, one adduct had a precursor ion peak at *m*/*z* 322.1492, which is nearly identical to the *m*/*z* 322.1600 DNA adduct observed in our study, although Guidolin et al. did not describe its chemical structure [31]. The MS/MS fragmentation data for both adducts are also highly similar (*m*/*z* 206.1122 and 162.0865 for *N*^1^-oxydiethylidene-dA, and *m*/*z* 206.1036 and 162.0773 for the unidentified adduct reported by Guidolin et al. [31]); these adducts are the same. Furthermore, this unidentified *m*/*z* 322.1 adduct was detected in human oral cell DNA, where its levels markedly increased after alcohol consumption and were notably higher than those of *N*^6^-ethyl-dA. This observation aligns with our findings from the mouse model. As noted earlier, CrPdG has been implicated in base substitution, suggesting that this novel *N*^1^-oxydiethylidene-dA adduct may also be mutagenic. To confirm whether *N*^1^-oxydiethylidene-dA acts as a driver adduct of SBS16, the further analysis of its mutagenicity and mutation pattern is required.

In recent years, several studies have conducted comprehensive analyses of the mutation patterns induced by AA using whole-genome analysis techniques. Thapa et al. analyzed the mutation signature induced by AA using a yeast gene reporter system, which allowed for the controlled generation of long single-stranded DNA regions [57]. Their findings revealed that AA-induced C:G to A:T transversions were predominant and occurred mainly at the TC/GA motif compared to those of the mock-treated controls. Additionally, AA treatment led to an excess of deletion events longer than four bases. Although the mutation signatures associated with AA showed slight differences from C to T, from C to A, and from T to C compared to those of the controls, they did not closely resemble any mutation signatures recorded in the COSMIC database. A similar result was reported by another group, demonstrating that AA induces strand-biased G to T transitions at a high frequency on ssDNA, particularly at the gCn to gAn motif, using a yeast genetic reporter system [58]. However, these results are derived from yeast ssDNA and may not accurately reflect in vivo conditions. In fact, AA has been reported to generate exocyclic etheno-type DNA adducts via lipid peroxidation [28]. 1,*N*^6^-ethenodeoxyadenosine (NεdA) may be formed in vivo through a mechanism similar to that of NεdG. It has already been reported that NεdA can introduce A to G mutations [59], suggesting that exocyclic adducts, such as *N*^1^-oxydiethylidene-dA and NεdA, may contribute to the formation of the SBS16 mutation pattern. To establish a definitive link between alcohol (or AA) exposure and SBS16, it is essential to conduct **in vivo** analyses using biologically derived samples.

Finally, although SBS16 is believed to be associated with AA exposure from alcohol consumption, this signature has also been observed in samples from patients with no history of alcohol exposure and has been linked to cigarette smoking or a combination of smoking and drinking habits [60,61,62,63]. However, since AA is produced endogenously in the human body and is also present in cigarette smoke, the relationship between SBS16 and AA cannot be ruled out. Additionally, SBS16 is known to be associated with a polymorphism of *ALDH2* (*rs671*). ALDH2 is an enzyme that metabolizes AA, but it is also involved in the metabolism of other substrates, including lipid peroxidation products, such as 4-hydroxynonenal and malonaldehyde [64,65,66], as well as endogenous aldehydes like glyoxal and methylglyoxal [67]. Further analysis focusing on these substances may be necessary to determine the precise etiology of SBS16.

## 5. Conclusions

The alcohol-related mutation signature, SBS16, is characterized by T:A to C:G mutations. Based on the strong strand bias on the transcribed gene, adenine adducts may contribute to A to G transition in this signature. Herein, we investigated the mutagenic activity of the candidate driver adduct, *N*^2^-ethyl-dA, using in vitro systems. In conclusion, we assessed the mutagenicity of *N*^6^-ethyl-dA using primer extension assays and the *supF* gene mutation test, revealing that this DNA adduct was not mutagenic in either system. To identify an alternative candidate as a driver adduct for SBS16, which is frequently observed in esophageal, liver, and stomach cancers in the Japanese population, we employed a DNA adductome approach. This analysis led to the identification of a novel adenine adduct, *N*^1^-oxydiethylidene-dA, in which two AA molecules are bound to an adenine base in the reaction mixture of CT-DNA and AA. Moreover, *N*^1^-oxydiethylidene-dA was detected in the livers of mice, and its levels increased after ethanol administration. Currently, the data regarding the mutagenic activity of *N*^1^-oxydiethylidene-dA is unavailable. However, CrPdG, a structurally similar adduct, has been implicated in base substitutions, suggesting that the novel *N*^1^-oxydiethylidene-dA is likely mutagenic. Although further analysis is required to confirm this, alcohol may contribute to SBS16 induction by forming AA-related adenine adducts such as *N*^1^-oxydiethylidene-dA.

## Figures and Tables

**Table 1:** Acetaldehyde (AA)-related deoxyadenosine (dA) adduct levels detected in livers of mice.

Treatment	*N*^1^-oxydiethylidene-dA (Adducts/10^8^ Nucleotides) ^1^	*N*^6^-Et-dA (Adducts/10^8^ Nucleotides)
Control	7.07 ± 2.11	3.80 ± 3.00
10% ethanol	56.2 ± 22.9	5.20 ± 2.72
10% ethanol + disulfiram	134.6 ± 10.1	9.06 ± 2.91

^1^ Adduct levels were calculated as 2′-dA equivalents.

## Data Availability

The original contributions presented in this study are included in the article/Supplementary Material. Further inquiries can be directed to the corresponding author.

## Supplementary Materials

The following supporting information can be downloaded at: https://www.mdpi.com/article/10.3390/biom15060878/s1 and https://doi.org/10.5281/zenodo.13471841, Figure S1: Variable loading plots and the MS/MS fragment data of DNA adducts contributing to AA exposure other than Adduct 42; Figure S2: DQF-COSY (a), HMQC (b) and HMBC (c) spectra of isolated Fr.2 and its chemical structure; Figure S3: MS/MS fragmentation patterns of Fr.5 and Fr.6; Figure S4: LC-ESI-MS chromatograms of authentic *N*^6^-ethyl-dA, CT-DNA + AA (Adduct 42), and mouse liver DNA samples.

## Abbreviations

AA	Acetaldehyde
ALDHs	Aldehyde dehydrogenases
SNPs	Single-nucleotide polymorphisms
dG	2′-Deoxyguanosine
CrPdG	*α*-S- and *α*-R-methyl-*γ*-hydroxy-1, *N*^2^-propano-2′-deoxyguanosine
*N*^6^-ethyl-dA	*N*^6^-Ethyldeoxyadenosine
*supF*	Escherichia coli tyrosine amber suppressor tRNA gene
LC	Liquid chromatography
HRAM-MS	High-resolution accurate mass spectrometry
HPLC	High-performance liquid chromatography
CT-DNA	Calf thymus DNA
PCA–DA	Principal component analysis–discriminant analysis
dR	Deoxyribose
2D	Two-dimensional
PI	Production
