# Supplementary figures and images for: Analysis of Novel DNA Adducts Derived from Acetaldehyde
Source: Biomolecules. 2025 Jun 16;15(6):878. doi: 10.3390/biom15060878 (PMC12190651; doi:10.3390/biom15060878)

## Figure S1

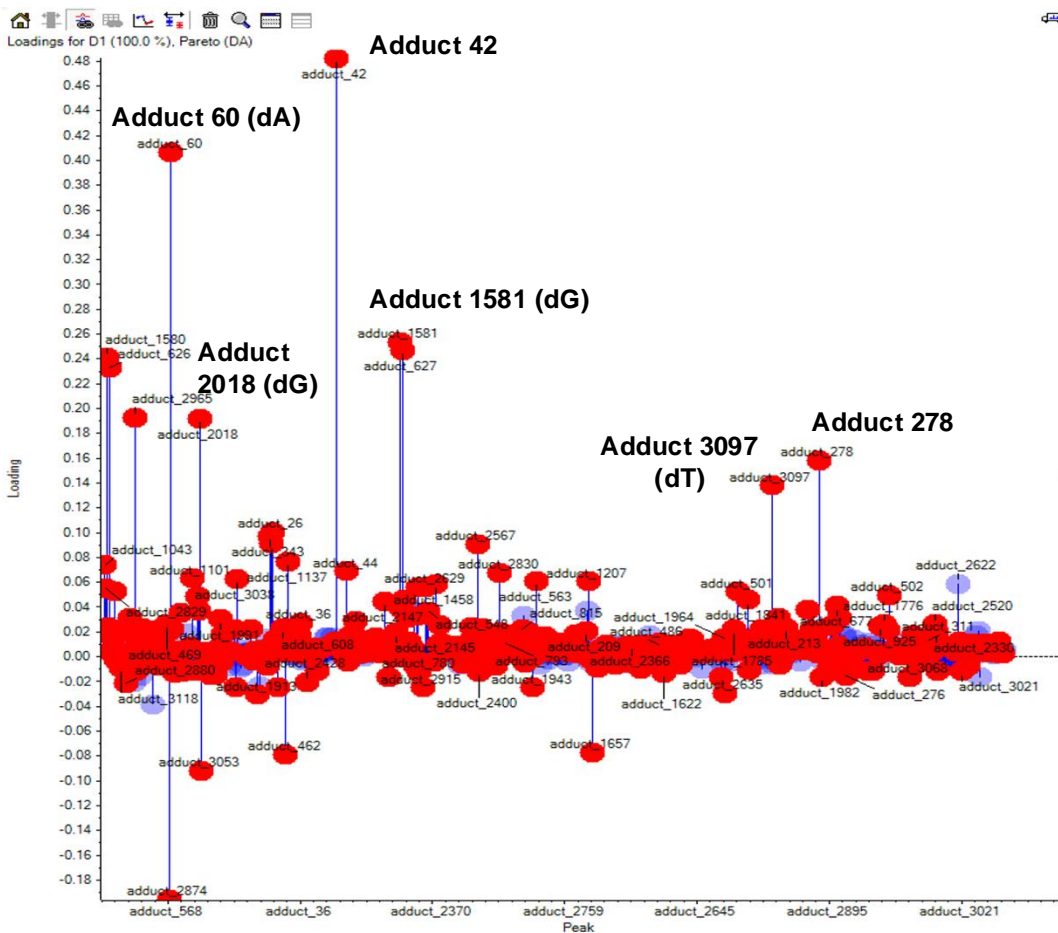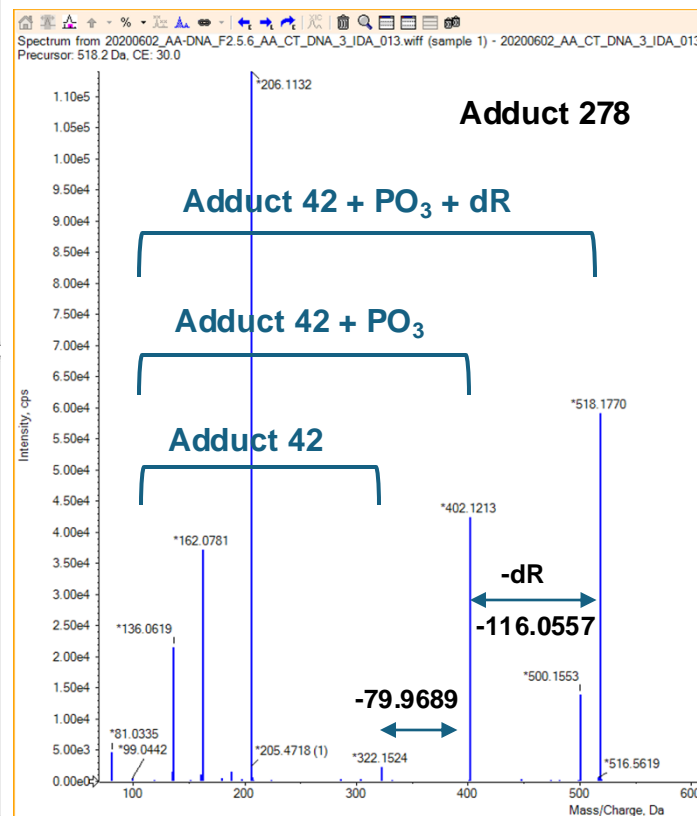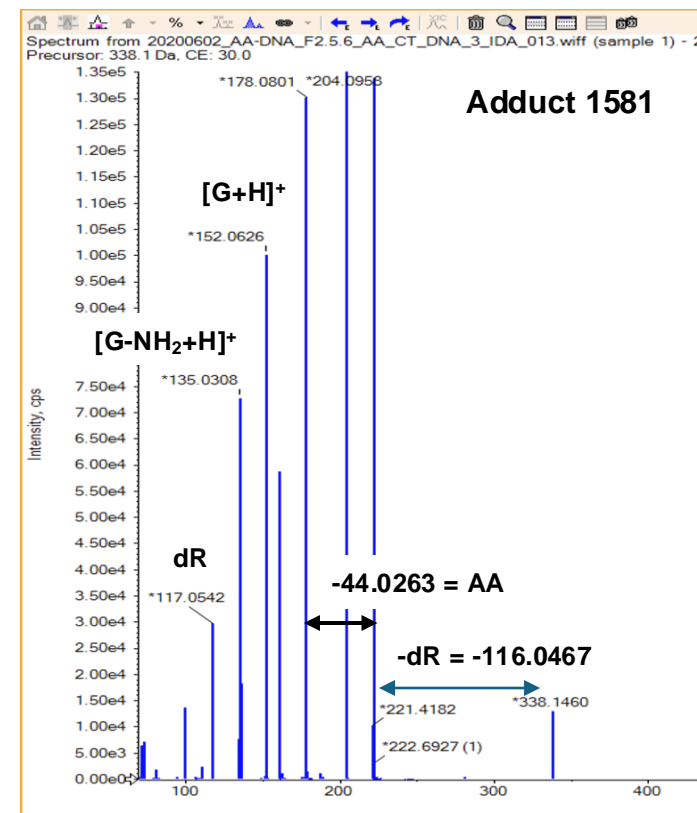

Supplement: Supplementary file 1 [file biomolecules-15-00878-s001.zip › biomolecules-3527361-supplementary/Supple Fig_rev1/Fig. S1_rev.pdf]

**a**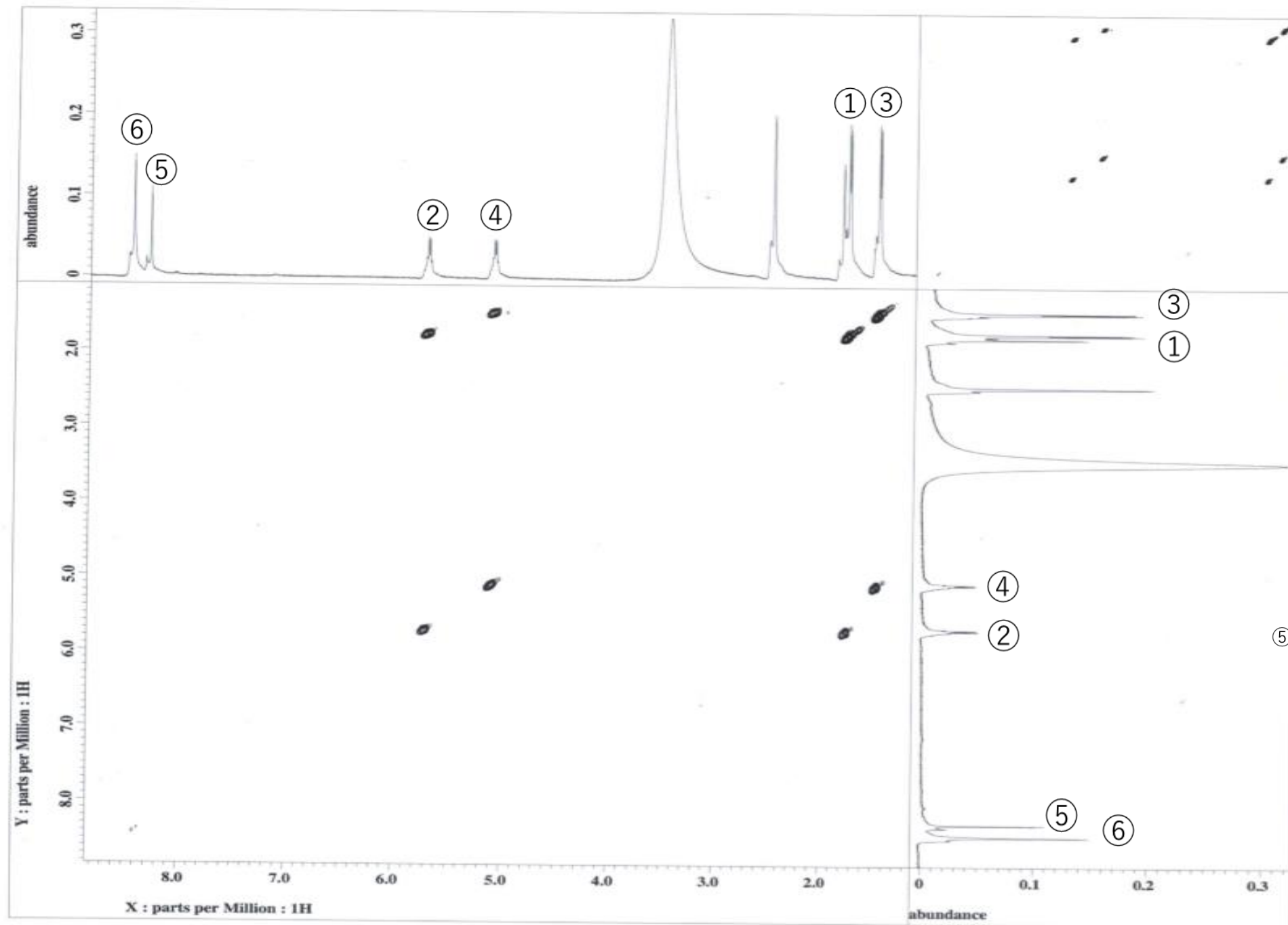**Figure S2**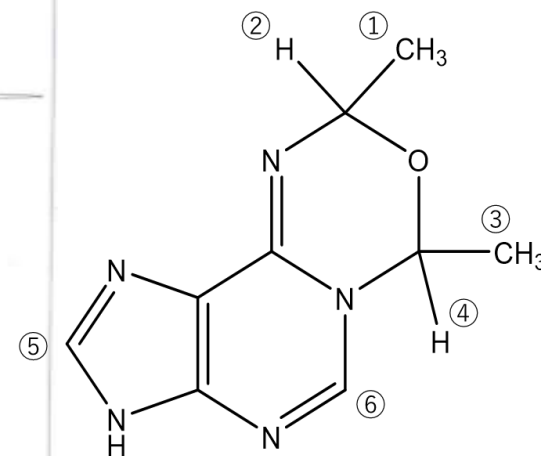

**b**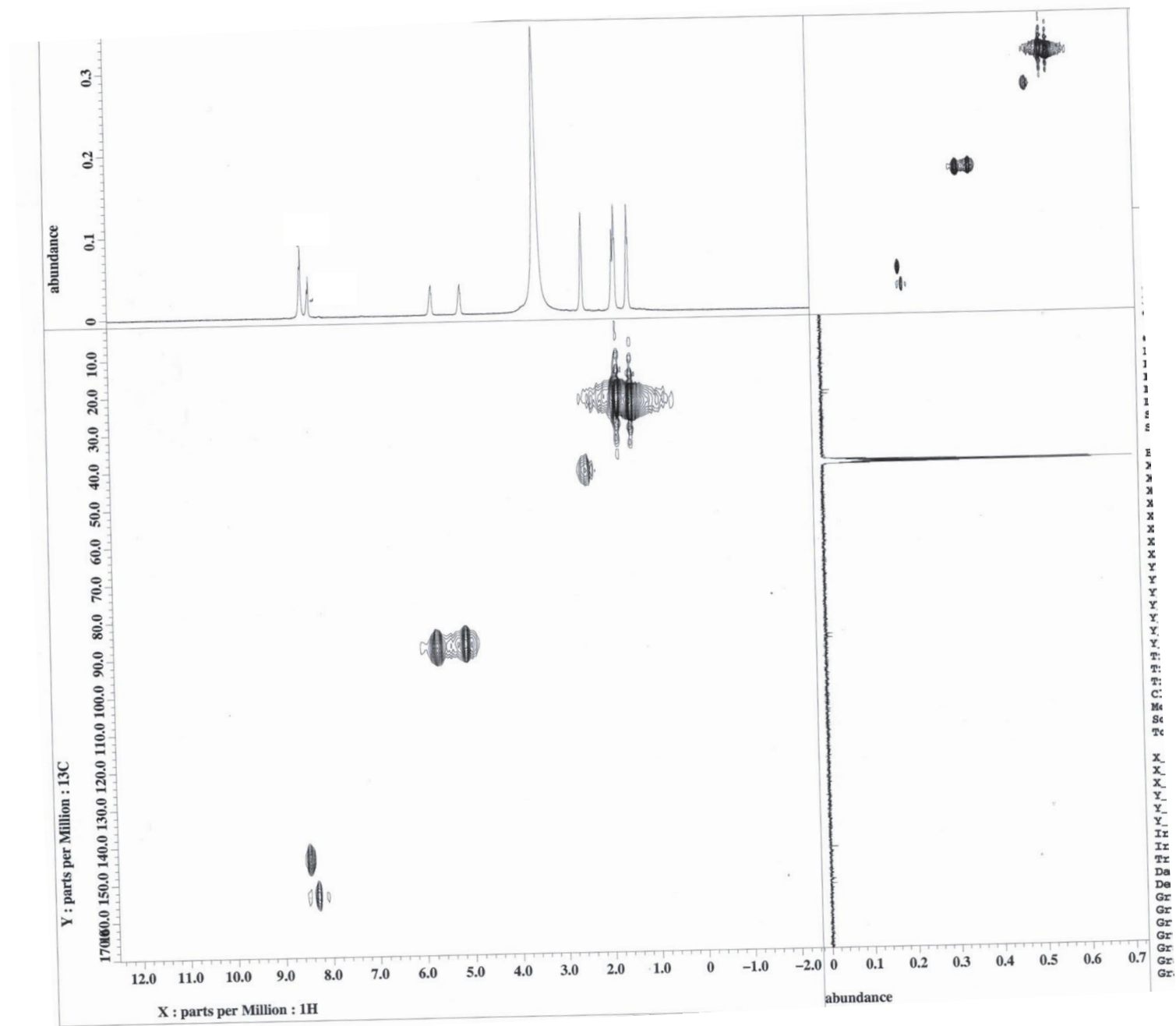

c

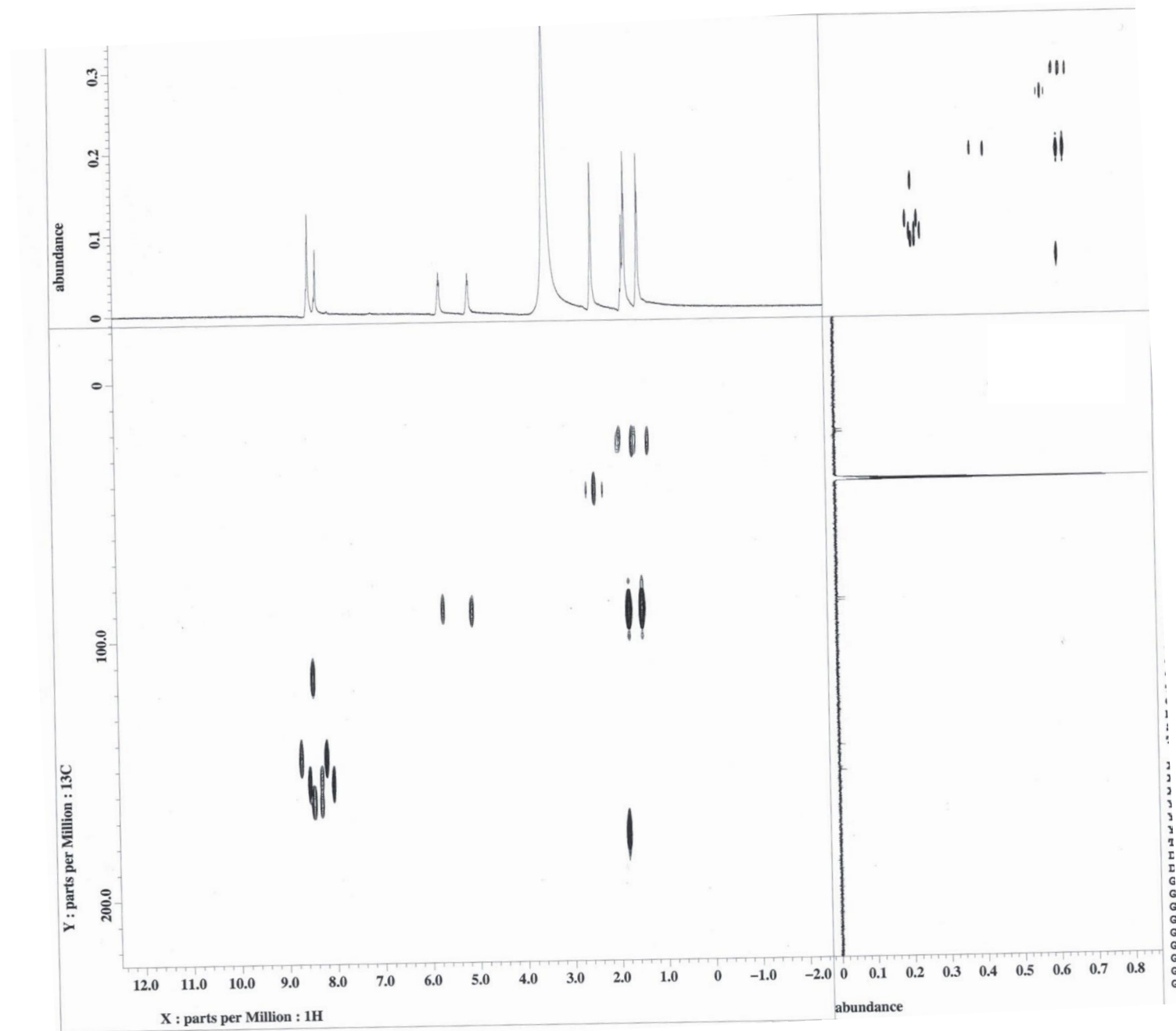

Supplement: Supplementary file 1 [file biomolecules-15-00878-s001.zip › biomolecules-3527361-supplementary/Supple Fig_rev1/Fig. S2_rev.pdf]

Figure S3

Fr. 5

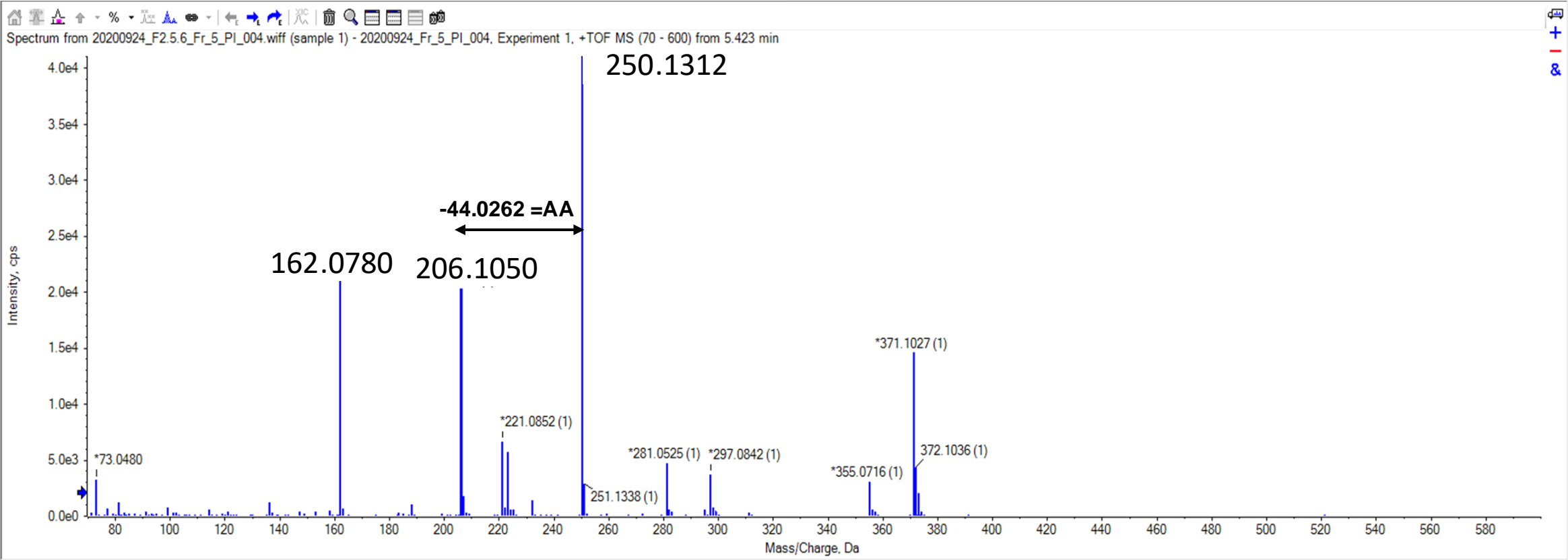

### Figure S3 (Continued)

## Fr. 6

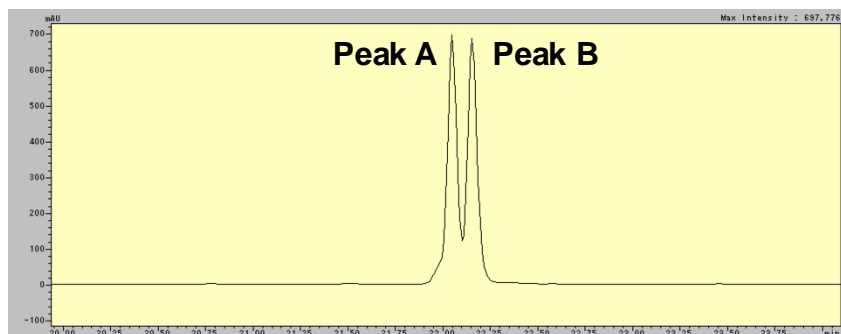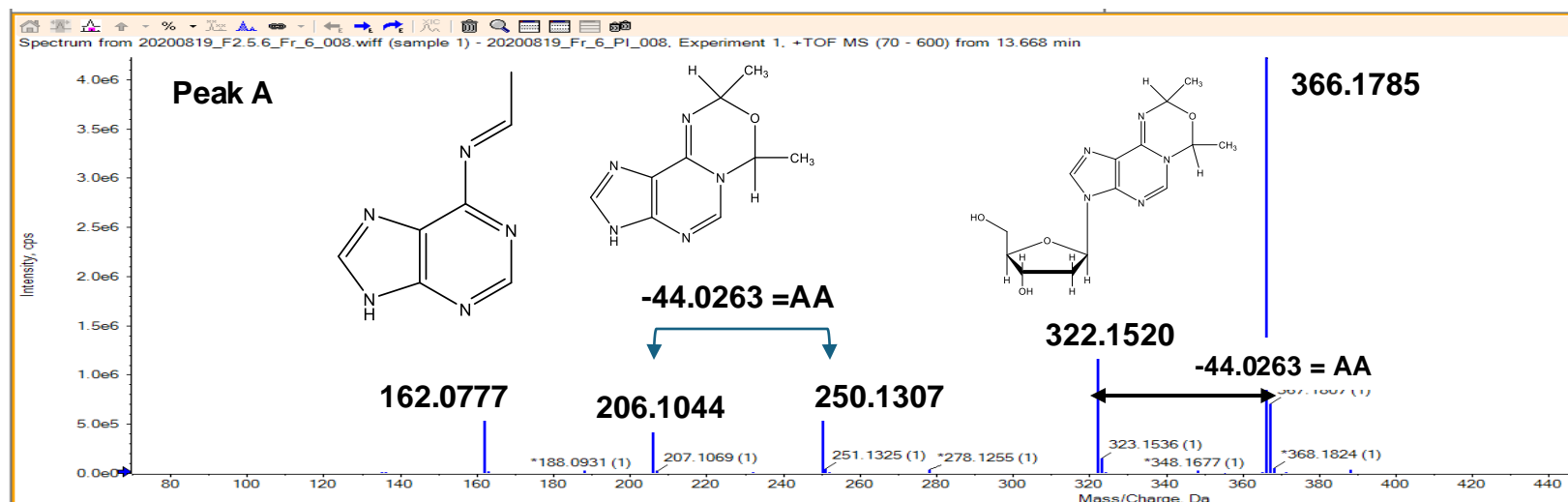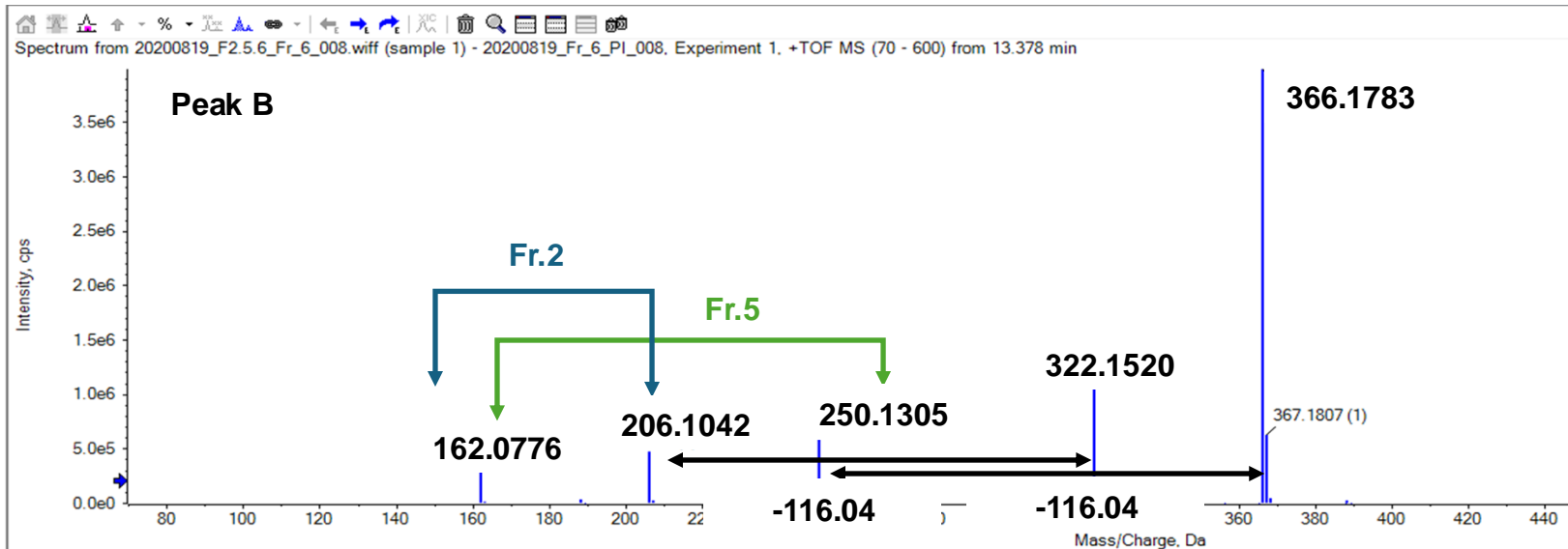

Supplement: Supplementary file 1 [file biomolecules-15-00878-s001.zip › biomolecules-3527361-supplementary/Supple Fig_rev1/Fig. S3.pdf]

Figure S4

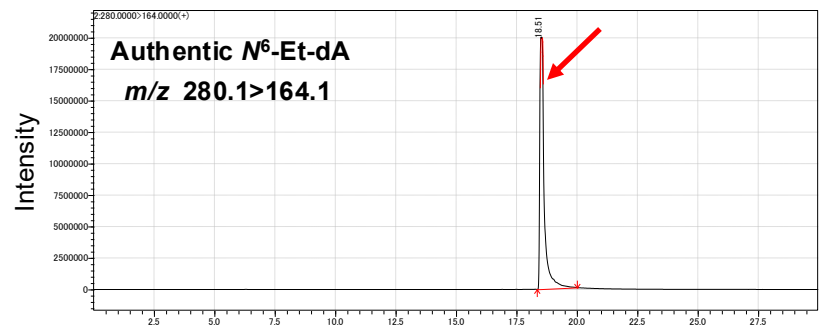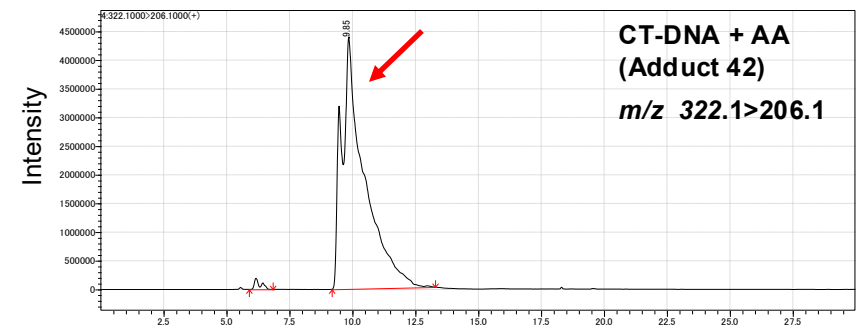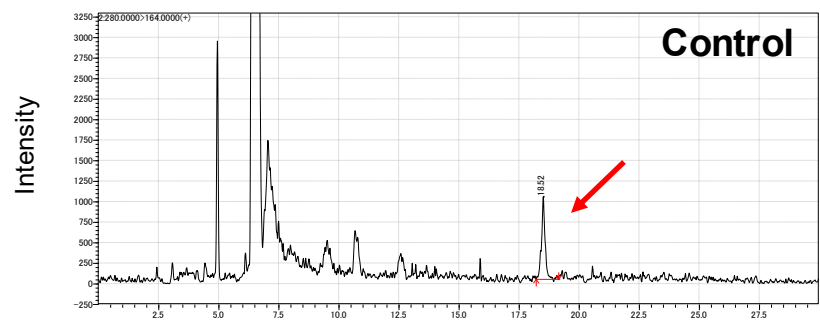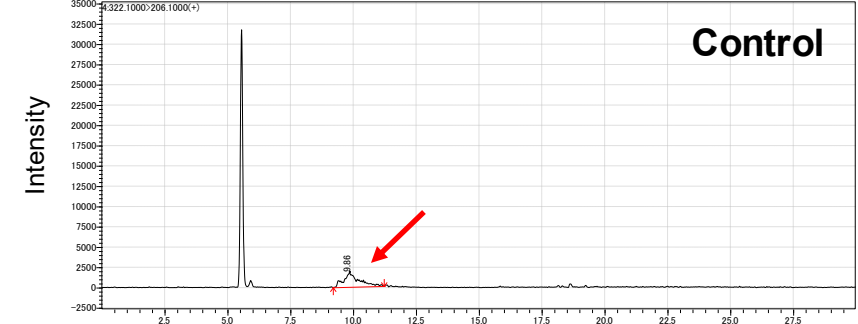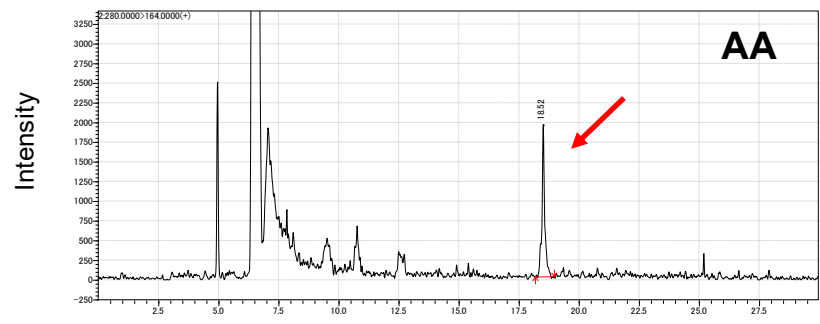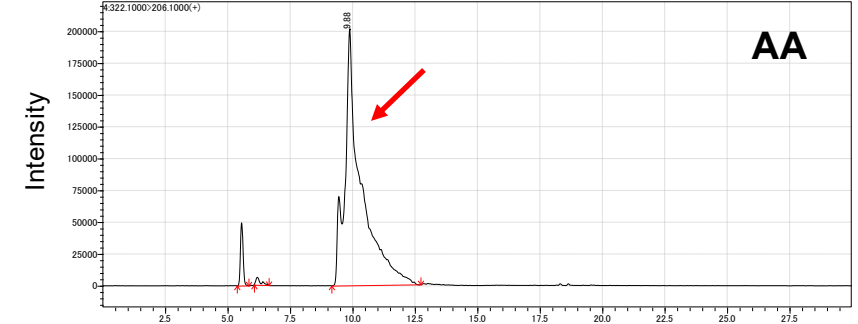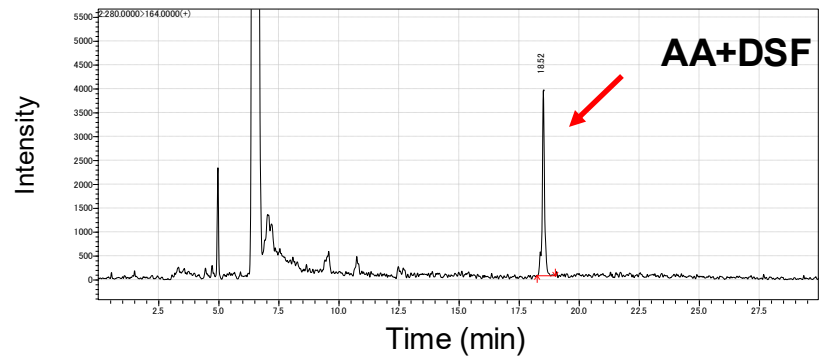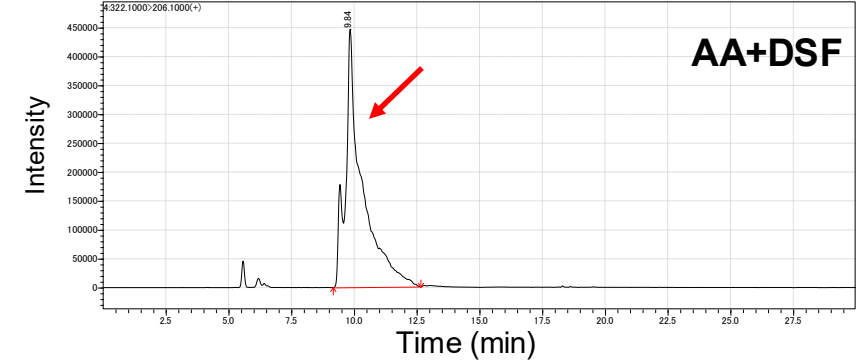

Supplement: Supplementary file 1 [file biomolecules-15-00878-s001.zip › biomolecules-3527361-supplementary/Supple Fig_rev1/Fig. S4.pdf]
